# Supplementary material for: NOX2 inhibition enables retention of the circadian clock in BV2 microglia and primary macrophages
Source: Front Immunol. 2023 Feb 6;14:1106515. doi: 10.3389/fimmu.2023.1106515 (PMC9939898; doi:10.3389/fimmu.2023.1106515)
Supplement: Supplementary file 1 [file DataSheet_1.docx]

**Supplementary Figures**

**NOX2 Inhibition Enables Retention of the Circadian Clock in BV2 Microglia and Primary Macrophages**

**Iswarya Muthukumarasamy^1,2^, Sharleen M. Buel^2,3^, Jennifer M. Hurley^2,3^, Jonathan S. Dordick^1,2,3*^**

^1^Chemical and Biological Engineering, Rensselaer Polytechnic Institute, Troy, NY, United States

^2^Center for Biotechnology & Interdisciplinary Studies, Rensselaer Polytechnic Institute, Troy, NY, United States

^3^Biological Sciences, Rensselaer Polytechnic Institute, Troy, NY, United States

***Correspondence:** Jonathan S. Dordick (dordick@rpi.edu)

**
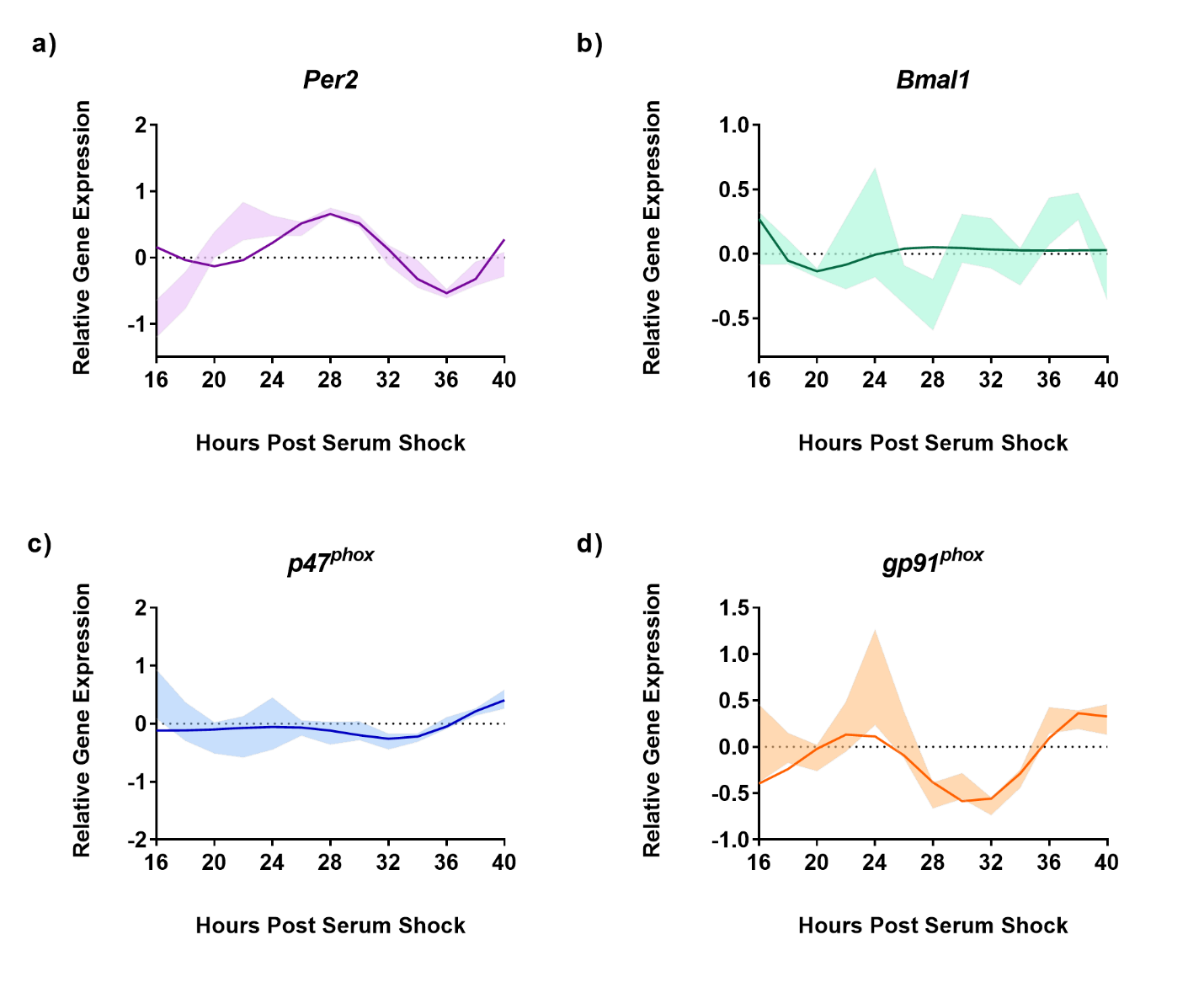
**

**Supplementary Figure 1. Inhibition of NOX2 by apocynin under LPS activation.** ECHO fitted plots for mRNA expression (n = 3) of clock genes (a) Per2 and (b) Bmal1, and NOX2 components (c) gp91^phox^ and (d) p47^phox^ in BV2 microglia in the presence of 1 μg/mL LPS and 25 μM GSK2895039. Data represented as fold change in expression using Hprt1 as a reference gene and HPS0 as a reference sample for the ΔΔCt method of data analysis. Bold line represent model fit with shaded region representing the standard deviation of model at each time point. All plots had p<0.05 for ECHO significance fit.


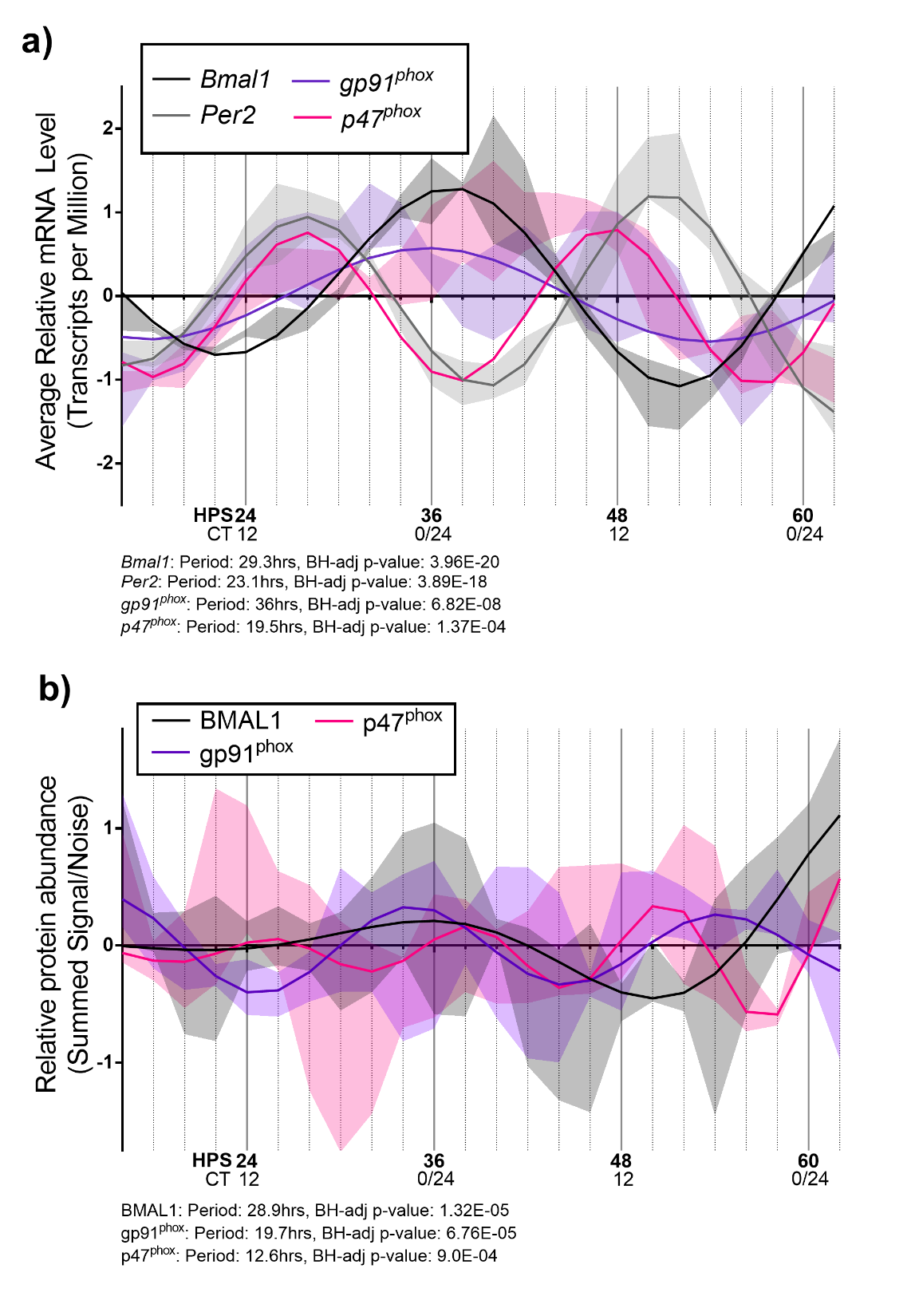


**Supplementary Figure 2. mRNA and protein levels of clock genes and NOX2 components show oscillations in mouse bone marrow-derived macrophages.** Data obtained from previously available RNA sequencing and proteomic analysis datasets showing (a) mRNA oscillations of Per2, Bmal1, p47^phox^ and gp91^phox^ reported in transcripts per million, (b) relative protein abundance oscillations of BMAL1, p47^phox^ and gp91^phox^. Bold line represent model fit with shaded region representing the standard deviation of model at each time point. All plots had p<0.05 for ECHO significance fit.


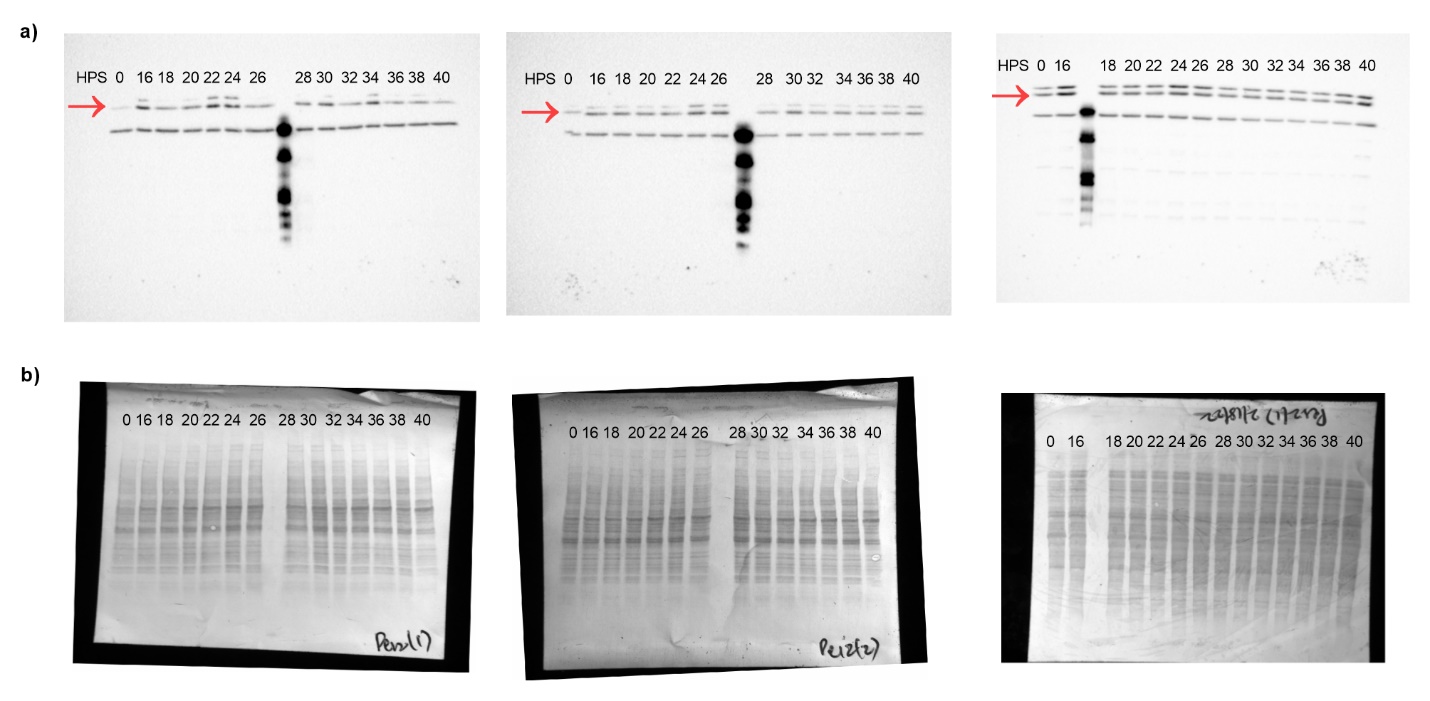


**Supplementary Figure 3. Complete western blot images corresponding to PER2 and its amido black stain in BV2 microglia.** (a) PER2 (MW:137 kDa) expression in BV2 microglia for three biological replicates measured every 2 h for 24 h starting at HPS16. (b) Amido black staining of the blots corresponding to PER2 expression in BV2 microglia for three biological replicates measured every 2 h for 24 h starting at HPS16.


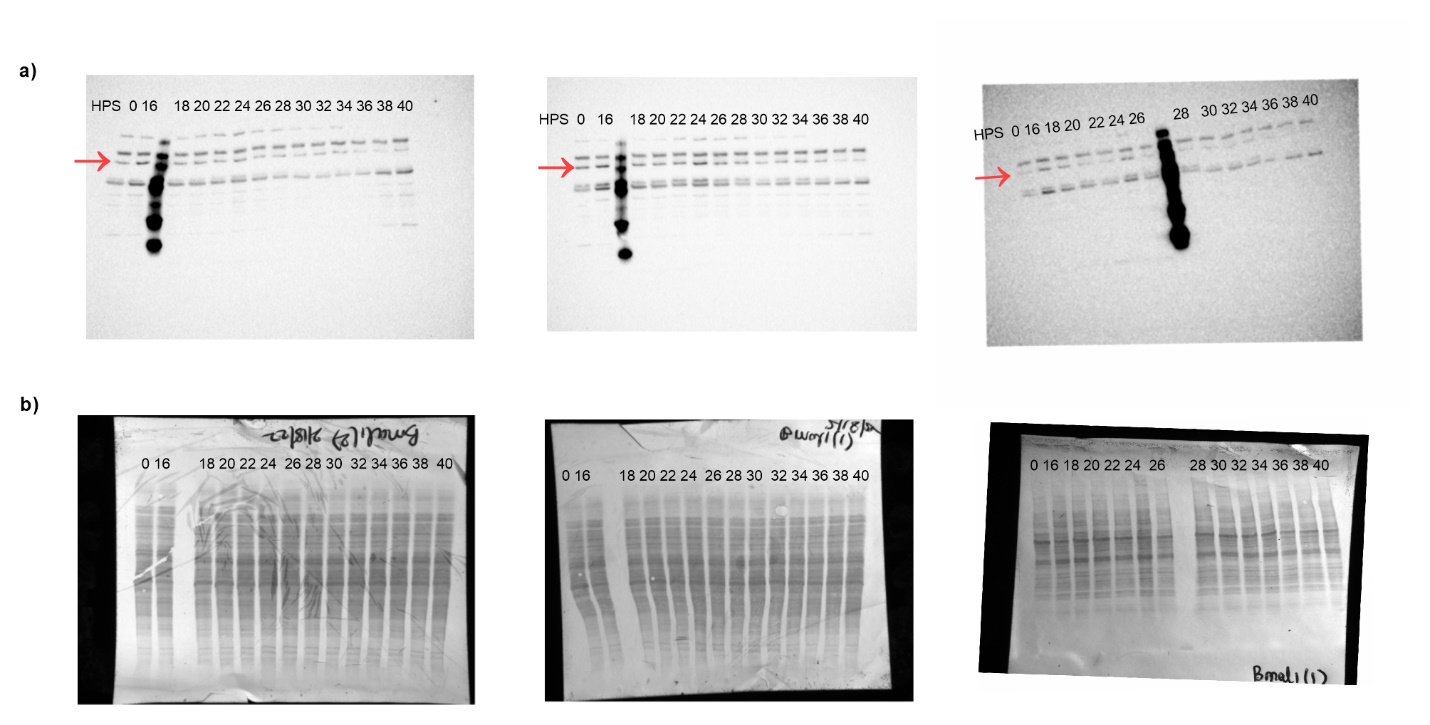


**Supplementary Figure 4. Complete western blot images corresponding to BMAL1 and its amido black stain in BV2 microglia.** (a) BMAL1 (MW: 69 kDa) expression in BV2 microglia for three biological replicates measured every 2 h for 24 h starting at HPS16. (b) Amido black staining of the blots corresponding to BMAL1 expression in BV2 microglia for three biological replicates measured every 2 h for 24 h starting at HPS16.


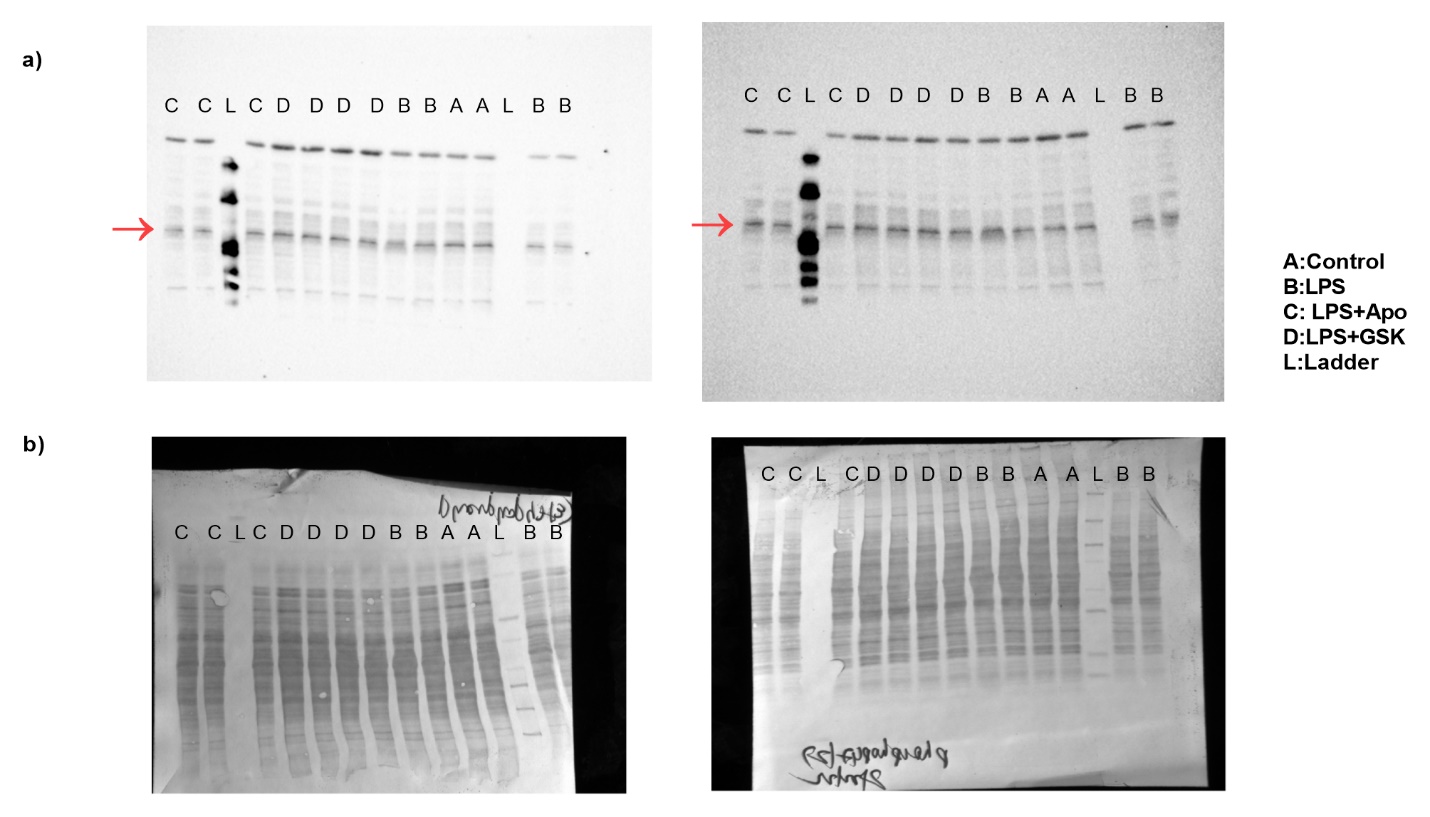


**Supplementary Figure 5. Complete western blot images corresponding to phosphor-p47^phox^(Ser370) and its amido black stain in BV2 microglia.** (a) Phosphorylated-p47^phox^ levels (MW: 45 kDa) measured in BV2 microglia with and without LPS and NOX2 inhibitors apocynin and GSK2795039, and IL-4 for three biological replicates. (b) Amido black staining of the blots corresponding to Phosphorylated-p47^phox^ levels measured in BV2 microglia with and without LPS and NOX2 inhibitors apocynin and GSK2795039, and IL-4 for three biological replicates.

**Supplementary Table 1. ECHO data for transcript and protein level analysis for clock genes and NOX2 components.** The compiled data including ECHO fitted values and ECHO replicate values for analysis of *Per2, Bmal1, p47^phox^* and *gp91^phox^* genes, and PER2 and BMAL1 protein levels in BV2 microglia.
